# The Importance of Pore-Forming Toxins in Multiple Organ Injury and Dysfunction

**DOI:** 10.3390/biomedicines10123256

**Published:** 2022-12-14

**Authors:** Simon T. Abrams, Lijun Wang, Jun Yong, Qian Yu, Min Du, Yasir Alhamdi, Zhenxing Cheng, Caroline Dart, Steven Lane, Weiping Yu, Cheng-Hock Toh, Guozheng Wang

**Affiliations:** 1 Department of Clinical Infection, Microbiology and Immunology, University of Liverpool, Liverpool L69 7BE, UK; 2 Coagulation, Liverpool University Hospitals NHS Foundation Trust, Liverpool L7 8XP, UK; 3 The Medical School, Southeast University, Nanjing 210009, China; 4 Department of Gastroenterology, The First Affiliated Hospital, Anhui Medical University, Hefei 230032, China; 5 Institute of Systems, Molecular and Integrative Biology, University of Liverpool, Liverpool L69 7ZB, UK; 6 Institute of Translational Medicine, University of Liverpool, Liverpool L7YB, UK; 7 Roald Dahl Haemostasis & Thrombosis Centre, Liverpool University Hospitals NHS Foundation Trust, Liverpool L78XP, UK

**Keywords:** pore-forming toxin, cell membrane integrity, extracellular histones, pneumolysin, sepsis, multiple organ injury, multiple organ dysfunction syndrome

## Abstract

Background: Multiple organ injury and dysfunction often occurs in acute critical illness and adversely affects survival. However, in patients who survive, organ function usually recovers without permanent damage. It is, therefore, likely that there are reversible mechanisms, but this is poorly understood in the pathogenesis of multiple organ dysfunction syndrome (MODS). Aims: Based on our knowledge of extracellular histones and pneumolysin, as endogenous and exogenous pore-forming toxins, respectively, here we clarify if the extent of cell membrane disruption and recovery is important in MODS. Methods: This is a combination of retrospective clinical studies of a cohort of 98 patients from an intensive care unit (ICU) in a tertiary hospital, with interventional animal models and laboratory investigation. Results: In patients without septic shock and/or disseminate intravascular coagulation (DIC), circulating histones also strongly correlated with sequential organ failure assessment (SOFA) scores, suggesting their pore-forming property might play an important role. In vivo, histones or pneumolysin infusion similarly caused significant elevation of cell damage markers and multiple organ injury. In trauma and sepsis models, circulating histones strongly correlated with these markers, and anti-histone reagents significantly reduced their release. Comparison of pneumolysin deletion and its parental strain-induced sepsis mouse model showed that pneumolysin was not essential for sepsis development, but enhanced multiple organ damage and reduced survival time. In vitro, histones and pneumolysin treatment disrupt cell membrane integrity, resulting in changes in whole-cell currents and elevated intracellular Ca^2+^ to lead to Ca^2+^ overload. Cell-specific damage markers, lactate dehydrogenase (LDH), alanine aminotransferase (ALT), and cardiac troponin I (cTnI), were released from damaged cells. Once toxins were removed, cell membrane damage could be rapidly repaired and cellular function recovered. Conclusion: This work has confirmed the importance of pore-forming toxins in the development of MODS and proposed a potential mechanism to explain the reversibility of MODS. This may form the foundation for the development of effective therapies.

## 1. Introduction

Multiple organ injury and multiple organ dysfunction syndrome (MODS) often occurs in critical illness, which includes sepsis, severe trauma, and severe pancreatitis [1,2,3]. Its development is associated with high mortality rates [4,5,6]. Unlike diseases, such as severe hepatitis or nephritis, the organ dysfunction can resolve without permanent damage to survivors [7]. MODS may be a spontaneous manifestation in patients with septic shock or disseminated intravascular coagulation (DIC), due to insufficient blood and oxygen supply to vital organs [8,9]. However, the majority of septic patients develop MODS without shock or DIC [10], and the underlying pathological mediators of multiple organ injury remain unclear.

Recently, damage-associated molecular patterns (DAMPs), such as extracellular histones, have been shown to promote thrombosis through the assembly of an alternative prothrombinase complex [11], as well as directly mediating multiple organ injury in critical illnesses [2,12,13]. Extracellular histones are the most abundant DAMPs and act as endogenous pore-forming toxins, through cell membrane binding, calcium (Ca^2+^) influx, membrane potential changes, and cytotoxicity to most vital organs [11,14,15,16]. Extracellular histones have also been demonstrated to activate Toll-like receptors (TLRs), including TLR-2, TLR-4, and TLR-9, and to mediate inflammatory responses [17,18].

Pathogen-associated molecular patterns (PAMPs), such as pneumolysin released from *Streptococcus pneumoniae*, also play an important role in multiple organ injury [19]. In a similar manner to extracellular histones, pneumolysin acts as an exogenous pore-forming toxin, leading to changes in membrane potential, Ca^2+^ influx, and even cell lysis [20,21]. Pore-forming toxins are commonly released by multiple species of bacteria [22], such as hemolysins from *Staphylococcus aureus* and *Escherichia coli*, listeriolysin from *Listeria monocytogenes*, and perfringolysin from *Clostridium perfringens* [23].

Ca^2+^ homeostasis is vital for cellular function and signaling, with nanomolar intracellular Ca^2+^ concentrations and millimolar extracellular Ca^2+^ concentrations being considered. This steep Ca^2+^ gradient is tightly maintained through cellular membrane integrity, alongside mitochondrial and endoplasmic reticulum control [24]. Disruption of cell membrane integrity and Ca^2+^ influx induced by pore-forming toxins will cause intracellular Ca^2+^ overload, a major pathological factor causing cell injury, dysfunction, and death [25,26].

On the other hand, Ca^2+^ influx and intracellular Ca^2+^ overload can trigger cellular membrane self-repair, which isolates the area of pore formation in the form of endosomes [27]. Damaged plasma membranes separated from the membrane wall will be either degraded intracellularly or released in the form of exosomes into the extracellular space. In this way, membrane integrity is restored [28]. If pore-forming toxins are cleared and no further damage ensues, the cellular and organ function recovers [29]. However, the persistence of pore-forming toxins at high concentrations will cause cell death, coagulopathy, and microcirculatory impairment, and it will adversely affect outcomes in patients with sepsis [30].

In this study, we will investigate whether cell membrane disruption by both endogenous (extracellular histones) and exogenous (pneumolysin) pore-forming toxins contribute to multiple organ injury and MODS in animal models and human diseases. We will clarify if this is a major pathological mechanism of multiple organ injury and dysfunction in critically ill patients.

## 2. Materials and Methods

### 2.1. Patients

A retrospective case-control study was performed on 98 patients consecutively admitted to the intensive care unit (ICU) at the Royal Liverpool University Hospital between June 2013 and January 2014. Data were obtained following written consent and ethical approval approved by the National Research Ethics Service Committee Northwest—Greater Manchester West and Liverpool Central (Ref: 13/NW/0089). Patients’ characteristics are presented in Table 1, including acute physiology and chronic health evaluation II (APACHE II) score [31] within 24 h and sequential organ failure assessment (SOFA) scores [32] from Day 1 to Day 4. The APACHE II score is an alternative validated tool for predicting patient mortality, whilst SOFA scores are mainly for determination of the extent of a person’s organ function or rate of failure. Therefore, association between circulating histones (Days 1–2 post ICU admission) and SOFA scores (Days 1–4 post ICU admission) were performed on the total cohort. These analyses were also performed on a subset of patients with no septic shock and no DIC (*n* = 64), where patients with shock alone (*n* = 20), DIC alone (*n* = 5), and shock+DIC (*n* = 9) were removed. 

### 2.2. Animal Models

C57/BL6 male mice from Beijing Vital River Laboratory Animal Technology were housed and used in sterile conditions at the Research Centre of Genetically Modified Mice, Southeast University, China. All procedures were performed according to state laws. C.Z.X and L.W hold the full animal licenses for using mice. All the animal models were created in our previous studies, and circulating ALT, cTnI, BUN, histones, and lung injury scores were measured, as described previously [2,13,15,20]. For survival time comparison, 8–10 mice per group were injected with D39 or PLN-A (4 × 10^7^ CFU i.p.; or 2 × 10^8^ CFU, i.v. lethal doses determined by pilot experiments). Dying mice were identified and euthanized by neck dislocation via close monitoring every 2–4 h after bacterial injection until all mice died. Intravenous or intraperitoneal administration was used to ensure the accuracy of bacterial doses given to mice.

### 2.3. Bacterial Culture

*Escherichia coli* K12, normal BL21(DE3) (New England Biolabs, Ipswich, MA, USA), and ClearColi BL21(DE3) (Cambridge Bioscience, Cambridge, UK), were cultured in LB broth (Melford, Ipswich, UK) in a 37 °C shaker at 180 rpm. *Streptococcus pneumoniae* D39 and PLN-A strains [20] were cultured in brain-heart infusion medium (Oxoid, Hampshire, UK) in a 37 °C incubator. All bacteria were harvested during logarithmic growth. Living bacteria were resuspended in saline for the determination of cloning formation units (CFU) and injection. For Western blotting, bacteria were lysed in clear lysis buffer (125 mM Tris, pH 6.8, 5 mM EDTA, 1% SDS and 10% glycerol).

### 2.4. Western Blotting

Bacterial lysates (50 µg proteins) and recombinant PLY (2 and 5 µg) [20] were subjected to SDS-PAGE and transferred onto immobilon-P PVDF membrane (Millipore, Watford, UK). After blocking, the membrane was probed with anti-PLY antibody (1:5000, Abcam, Cambridge, UK) and anti-mouse-HRP (1:10,000, Santa Cruz, Dallas, TX, USA). Bands were visualized using enhanced chemiluminescence (ECL).

### 2.5. Histones and PLY Treatment 

Cells (5 × 10^4^) were seeded in 96-well culture plates and cultured for 24 h in 5% CO_2_ at 37 °C until confluent. Histones (0–300 µg/mL, Sigma-Aldrich, Cambridge, UK) or PLY (0–3 µg/mL, produced in house) [20] were used to treat cells for one hour. The culture medium was collected and immediately centrifuged 200× *g*, 5 min. Cellular supernatants were stored at −80 °C for lactate dehydrogenase (LDH, Sigma-Aldrich, Cambridge, UK), alanine aminotransferase (ALT, Colorimetric, Abcam, Cambridge, UK), and cardiac troponin I (cTnI, RayBiotech, Peachtree Corners, GA, USA) quantification, as per the manufacturer’s instructions. Adherent cells were immediately washed twice with PBS and cultured in medium containing WTS-8 for 2 h and measured the absorbance (460 nm) for determining cell viability, as described previously [20].

### 2.6. Cell Culture

Human endothelial cell line (EAhy926, ATCC) and normal human liver cells (HL-7702, ATCC) were routinely cultured. Murine cardiomyocytes (HL-1, Prof WC Claycomb, Louisiana State University Medical Centre, USA) were cultured in Claycomb medium, as previously described [12]. Once fully confluent, HL-1 cells were spontaneously contracted at a rate of 5–6 Hz at 37 °C.

### 2.7. Confocal Microscopy

PLY-FITC were produced using FluoroTag™ FITC Conjugation Kit (Sigma-Aldrich, Cambridge, UK) and purified by gel filtration column (Thermo Fisher Scientific, Waltham, MA, USA). HL-1 cells cultured in a 35 mm glass bottom dish (Greiner Bio-one, Gloucestershire, UK) were preloaded with 5 μM Fluo-2AM (Thermo Fisher Scientific, Waltham, MA, USA). Five µg/ml FITC-PLY were added to the culture. Both intracellular Ca^2+^ and cell membrane-associated PLY-FITC were recorded using time lapse confocal microscopy (LSM 710, Zeiss) [33] in a maintained environment of 5% CO_2_ at 37 °C.

### 2.8. Measurement of Intracellular Ca^2+^

Intracellular calcium concentration [Ca^2+^]i was determined by measuring fluorescence emission at 510 nm during excitation at 340 nm and 380 nm, according to published protocols, with Fura-2AM as the fluorescent probe using a Hitachi F-7000 fluorescent spectrometer [20]. [Ca^2+^]i was calculated using the software provided.

### 2.9. Electrophysiology

Whole-cell currents were recorded using the perforated patch configuration from single EAhy926 cells using an Axopatch 200B amplifier (Axon Instruments, East Hawthorn, VIC 3123 Australia), as previously described [34].

### 2.10. HL-1 Cardiomyocyte Contractility

HL-1 cardiomyocyte contractility traces were recorded using a video edge-recognition system (IonOptix, MyoCam-S, Dublin, Ireland), as previously described [20].

### 2.11. SPR Assay

Egg L-α-phosphatidylcholine (PC), egg L-α phosphatidylethanolamine (PE), and brain (porcine) L-α-phosphatidylserine (PS) in powder form and diacylglycerol (1–2-dioleoyl sn-glycerol, DG) in chloroform were purchased from Avanti Polar Lipids (Alabaster, AL, USA). The powders were resuspended in chloroform, then liposome solutions (PM) consisting solely of PC, PE, and PS were freshly prepared in a 40:40:20 ratio, reflecting those in the plasma membrane. The C1 sensor surface for Biacore X100 was prepared by permanently immobilising a mixture of histones H3 and H4 (New England Biolabs, Ipswich, MA, USA) to cell 2 (active cell) and S100P protein to cell 1 (control cell), as described previously [11]. Binding curves were generated using PM and DG.

### 2.12. Statistical Analysis

The data from in vitro experiments and animal models are presented as means ± SD. An ANOVA test was used for the comparison of more than two groups and followed by the Student-Newman-Keuls test. Human data are presented as median and interquartile ranges (1st, 3rd quartiles). Correlation between circulating histones and organ injury markers utilized Spearman’s rank test. The Logrank test was used for comparison of survival rates. All analyses were performed using IBM SPSS Statistics for Windows, version 26 (IBM Corp., Armonk, NY, USA), and a *p* value (two-tailed) <0.05 was considered statistically significant.

## 3. Results

### 3.1. Association of Circulating Histones with SOFA Scores in Critically Ill Patients without Septic Shock or Severe Coagulopathy

It is well documented that circulating histones are strong procoagulants and important mediators of MODS in sepsis. We retrospectively analyzed the association between levels of circulating histones and SOFA scores in a cohort of 98 critical ill patients (Table 1) admitted to the intensive care unit (ICU). We found strong correlations, particularly between circulating histone levels on days 1 and 2 and SOFA scores on days 3 and 4 after admission to the ICU (Table 2), suggesting a potential causal–effect relationship of circulating histones with MODS in critical illnesses. Day 1 histone levels (*p* = 0.002) and SOFA scores were significantly (*p* < 0.001) higher in patients with septic shock and/or DIC (*n* = 34, Median circulating histones 27.06 µg/mL [Q1:5.04-Q3:47.63]; median SOFA score 11.0 [Q1:9.25-Q3:13.0]) in relation to those without shock and/or DIC (*n* = 64), median circulating histones 8.66 µg/mL [Q1:2.04-Q3:21.65]; median SOFA score 6.0 [Q1:3.0-Q3:9.0]). This demonstrated that high levels of circulating histones are associated with shock and coagulopathy. After removing patients with shock and/or DIC, subsequent correlation analysis still demonstrated strong correlations between circulating histone levels on day 1 and day 2 after admission, as well as SOFA scores on day 3 and day 4 (Table 2). These data support that circulating histones can directly induce organ damage independently of inducing coagulopathy, and their pore-forming property may play an important role in these patients.

### 3.2. Correlation of Circulating Histones with Organ Injury Markers in Animal Models

**Figure 1:**
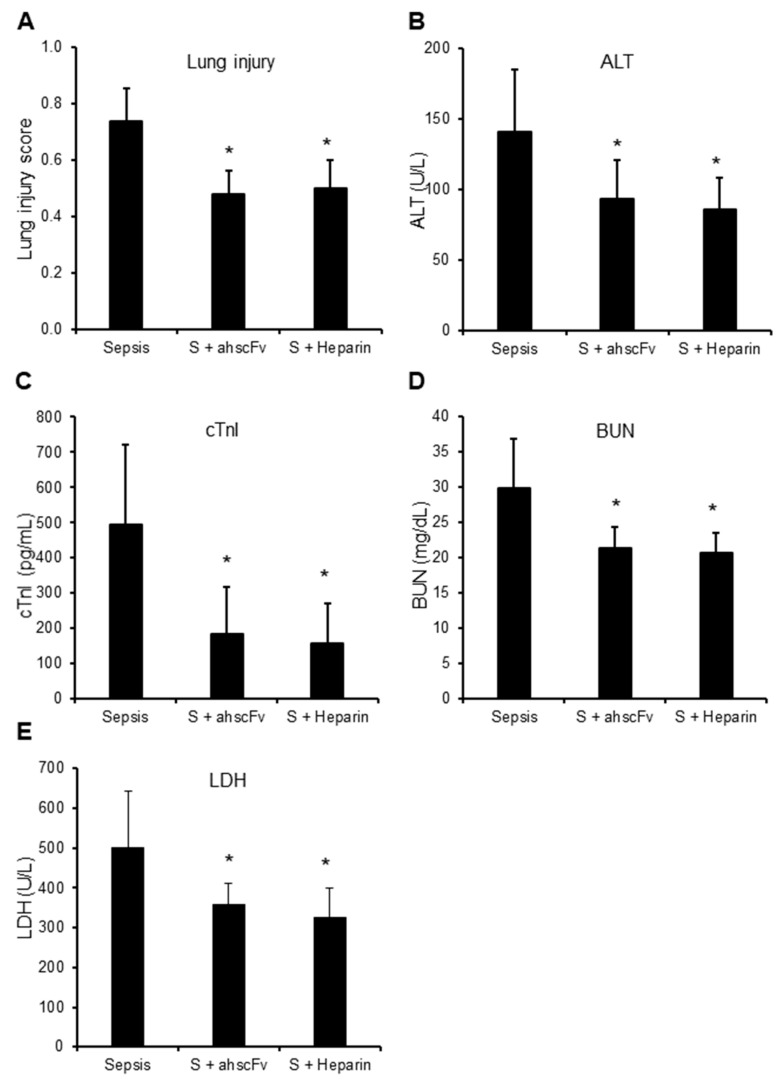
Anti-histone reagents reduce multiple organ injury. Sepsis was induced by injecting five mice per group with *E. coli* BL21 (1 × 10^8^ CFU per mouse) without (Sepsis) or with anti-histone scFv (S + ahscFv, 50 mg/kg) or non-anticoagulant heparin (S + Heparin, 50 mg/kg, Sigma-Aldrich) subcutaneous injection at 2, 8, 14, and 20 h after bacterial injection. Blood and organs were collected at 24 h after bacterial injection. Means ± SD of lung injury scores (**A**), blood ALT (**B**), cTnI (**C**), BUN (**D**), and LDH (**E**) were compared. An ANOVA test * *p* < 0.05 was compared to the sepsis group without anti-histone reagents. No statistical difference was found between ahscFv and heparin treatment groups.

In mouse trauma models, elevated circulating histones strongly correlated with raised circulating LDH levels (Table 3). Since circulating histones are released mainly from local tissue injury in trauma models [2], it is arguable that high circulating LDH was released by active secretion or from these damaged cells, rather than due to the pore-forming property of histones. However, the significant elevation of organ-specific markers, including cTnI and ALT, indicate the presence of cell leakage caused by pore-formation. Increased BUN and lung injury scores indicated multiple organ injury. The strong correlation of these markers to circulating histones (Table 3) suggest that elevated circulating histones are important mediators of multiple organ injury and dysfunction, and their pore-forming property played important roles. In sepsis mouse models, we also observed similar changes to the trauma models (Table 3). Both anti-histone scFv and non-anti-coagulant heparin significantly reduced lung injury, ALT and cTnI leakage, BUN, as well as LDH levels (Figure 1A,E), which further confirmed the importance of histone-mediated cell injury.

### 3.3. Infusion of Histones or PLY in Mice Causes Cell Leakage and Multiple Organ Injury

**Figure 2:**
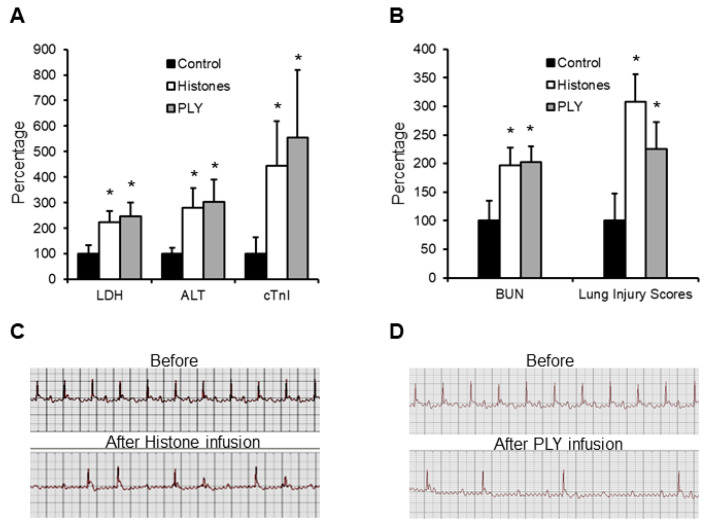
Calf thymus histones and PLY infusion mouse models. Five mice per group were infused with saline (control), calf thymus histones (50 mg/kg), or PLY (400 µg/kg) through tail veins. Blood was collected at 12 h and LDH, ALT, cTnI (**A**), as well as BUN and lung injury scores (**B**), were detected. The means ± SD of the relative increase to controls are presented. An ANOVA test * *p* < 0.05 was compared to the control. ECG was recorded before and within 4 h after histone (**C**) or PLY (**D**) infusion. Typical traces showing arrythmia are presented.

Circulating histones released from host cells can be regarded as endogenous pore-forming toxins, whilst the pore-forming toxins released by bacteria are considered as exogeneous. In sepsis, both endogenous and exogeneous pore-forming toxins could coexist, depending on the bacterial strains. PLY released by *Streptococcus pneumoniae* is common, and its toxicity to cardiomyocytes has been demonstrated in our previous study [20]. In this work, we used either histones (50 mg/kg) or PLY (400 µg/kg)-infusion mouse models and found that circulating LDH, ALT and cTnI were elevated (Figure 2A), indicating that histones and PLY caused leakage of multiple cell types. BUN and lung injury scores were also significantly increased (Figure 2B), indicating multiple organ injury. No significant difference between histones and PLY infusion was observed. Using ECG to monitor cardiac rhythm, and cardiac arrhythmia occurred in over 50% of mice infused with either histones or PLY (Figure 2C,D). These data strongly support that pore-formation on cell membranes is pivotal to the manifestation of MODS.

### 3.4. PLY-Deletion in Bacteria Does Not Inhibit Sepsis-Induction but Reduces Cell Leakage and Increase Mouse Survival

**Figure 3:**
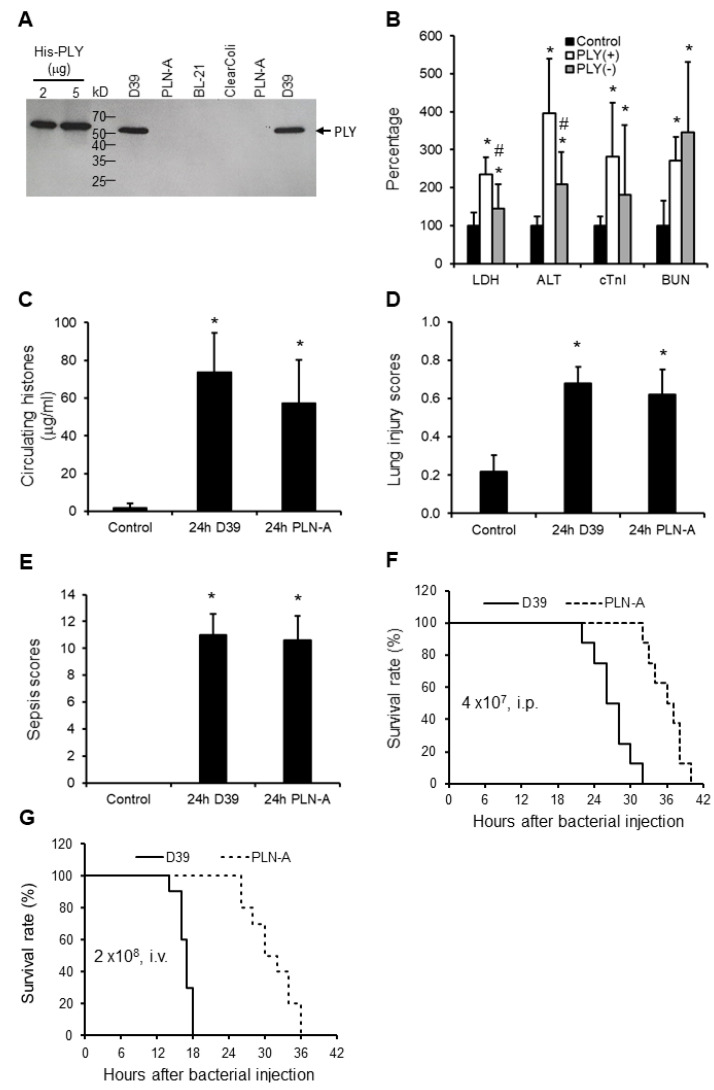
Mice sepsis models induced by peritoneal injection of D39 and PLN-A bacteria. (**A**) Western blot shows that D39 has detectable PLY, but PLN-A or *E.Coli* have no detectable PLY. (**B**) Five mice per group were injected with saline (control), D39 (PLY(+)) (4 × 10^7^ CFU/mouse, i.p.), or PLN-A (PLY(−) (4 × 10^7^ CFU/mouse, i.p.). Blood and organs were collected at 24 h after injection. The means ± SD of relative increase in LDH, ALT, cTnI, and BUN are presented. An ANOVA test * *p* < 0.05 was compared to the control. # *p* < 0.05 was compared to the PLY(+) bacteria-induced mouse model. The means ± SD of circulating histones (**C**), lung injury scores (**D**), and sepsis scores (**E**) showed no statistical difference between PLY (+) and PLY (−) models, but * *p* < 0.05 was compared to control groups. (**F** and **G**) Survival curves of mice injected with D39 or PLN-A (4 × 10^7^ CFU/mouse i.p. (**F**); or 2 × 10^8^ CFU/mouse i.v. via the tail vein (**G**)). 8–10 mice per group, log rank test, *p* < 0.05.

The presence of PLY was confirmed in *S. pneumoniae* serotype 2 strain (D39), but not in the PLY-deficient isogenic strain (PLN-A), *E.Coli* BL-21 or the ClearColi BL-21 (Figure 3A). LDH and ALT were significantly higher in D39-infected (PLY^+ve^) mice compared to PLN-A-infected (PLY^−ve^) mice (Figure 3B), indicating that PLY plays important roles in cellular leakage. Circulating histone levels are high in both models (Figure 3C). However, no statistical significance was observed in cTnI, BUN, histone levels (Figure 3B,C), lung injury scores (Figure 3D), or sepsis scores (Figure 3E) between D39-infected and PLN-A-infected mice. These data suggest that other toxic factors, such as circulating histones, may be more important in mediating multiple organ injury in these models. In contrast, mice infected (i.p.) with D39 died earlier than mice injected with PLN-A (Figure 3F). A similar pattern was observed via intravenous injection (i.v.) of these bacteria (Figure 3G).

### 3.5. Endogenous and Exogenous Pore-Forming Toxins cause Ca^2+^ Overload to Stress Cells In Vitro

**Figure 4:**
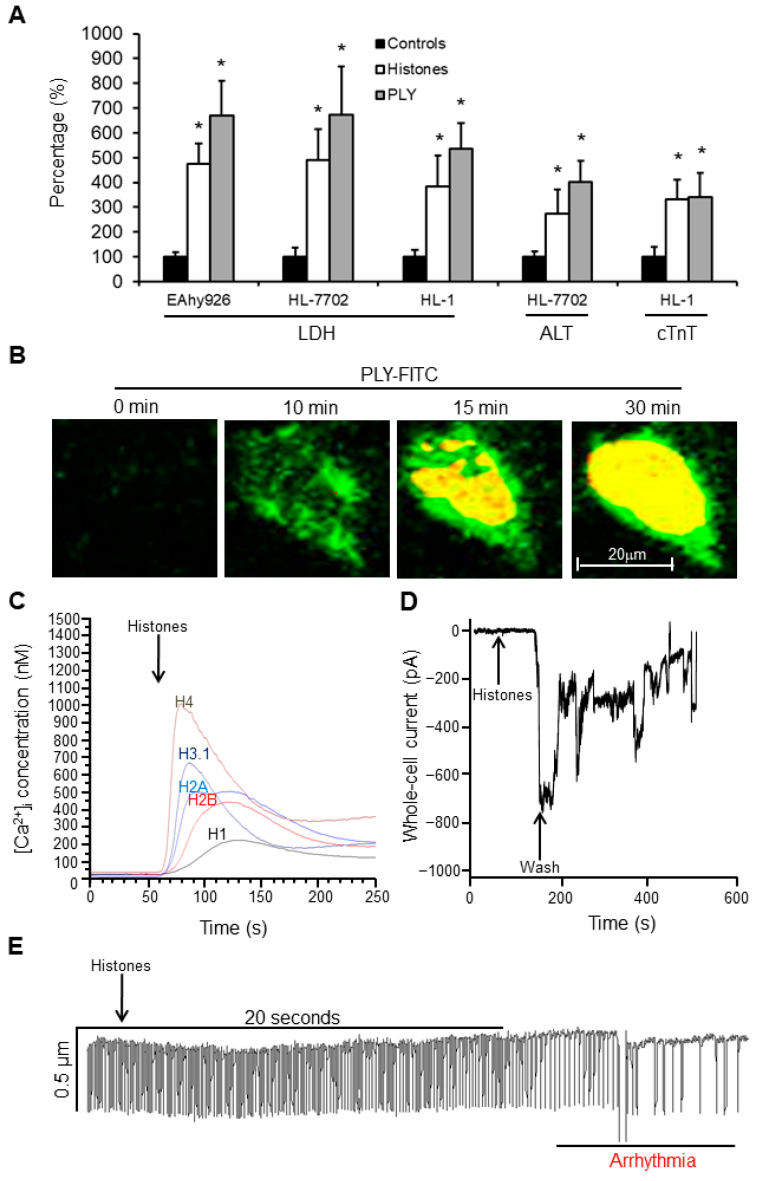
Extracellular histones and PLY interrupt cell membrane integrity. The concentrations of histones and PLY and the time courses to treat different cell lines were determined by monitoring cell viability. (**A**) Cultured cells were treated with 100 µg/mL calf thymus histones or 1.5 µg/mL PLY for 1 h (no significant effect on cell viability was monitored by WST-8 assay under these culture conditions), and the medium was collected. LDH activity in all three cell lines (EAhy926, HL-7702, and HL-1 cells) was measured, and the means ± SD of relative increase were obtained by setting the control as 100% from three independent experiments. Similarly, ALT from HL-7702 liver cells and cTnI from HL-1 cardiomyocytes were measured and presented. * *p* < 0.05, and Student’s *t*-test was compared and treated to the control. (**B**) Typical images of FITC-PLY (5 µg/mL, green) in cultured medium (with 2 mM CaCl_2_) of HL-1 cells preloaded with Fura-2 AM. After FITC-PLY concentrated on cell membrane, the calcium entered the cells and induced fluorescence of Fura-2AM (yellow). Bar = 20μm. (**C**) Individual histones (New England Biolab) were added to the culture medium of EAhy926 cells preloaded with Fura-2 AM. Intracellular Ca^2+^ concentrations were monitored and calculated using a Hitachi F-7000 fluorescent spectrometer. (**D**) Typical trace of whole-cell current changes of single EAhy926 cells upon calf thymus histone (20 µg/mL) treatment and washing using an Axopatch 200B amplifier. (**E**) Typical HL-1 cell contraction trace after treatment with calf thymus histones (75 µg/mL) using a video edge-recognition system.

Endothelial cells (EAhy926), liver cells (HL-7702), and cardiomyocytes (HL-1 cells) were treated with endogenous (histones) and exogeneous (pneumolysin [PLY]) pore-forming toxins. We found that both histones and PLY caused significant release of LDH, ALT, and cTnI in a dose-dependent manner. Using low doses of toxins, we still observed release of these markers without cell death, strongly indicating cellular leakage (Figure 4A). Using FITC-PLY to treat cultured HL-1 cells preloaded with calcium indicator, PLY concentrated on the cell membrane within 10 min and induced increases in intracellular Ca^2+^ (Figure 4B). Similarly, we found increased intracellular Ca^2+^ following treatment of endothelial cells with individual recombinant human histones (Figure 4C). Histones H4 and H3 induced greater intracellular Ca^2+^ increases compared to other histones (H1, H2A and H2B). EAhy926 cells treated with histones showed increases in whole-cell current (Figure 4D), and means ± SD were −722.33 ± 86.63pA from three independent experiments. Following washing to remove histones, these inward cellular currents gradually returned back to baseline to indicate membrane self-repair. Functional analysis demonstrated that histone treatment disturbed the auto-rhythm of HL-1 cardiomyocytes (Figure 4E), and the auto-rhythm recovered immediately after histone removal (data not shown).

### 3.6. Modelling Histone–Phospholipid Interaction, Pore Formation, and Self-Repairing Mechanisms

**Figure 5:**
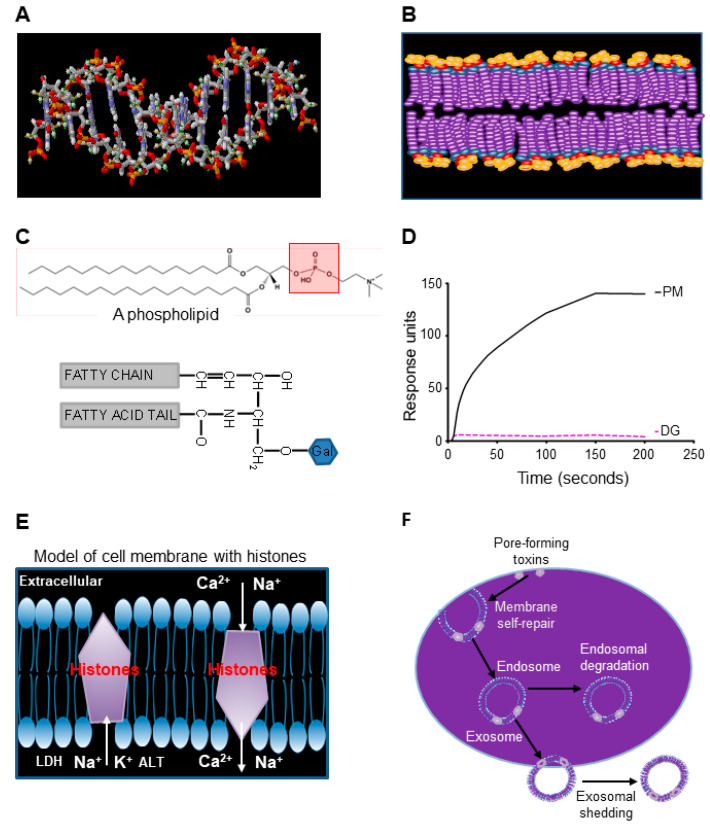
Extracellular histones interact with phospholipids to interrupt membrane integrity. (**A**) and (**B**). Comparison of phosphate groups (red) on double-stranded DNA (A) and cell membrane (B). Both double-stranded DNA and cell membrane have bilayers of phosphate groups. (**C**) Top panel shows the basic structure of phospholipid (phosphatidylcholine as an example) containing a phosphate group (red square). The lower panel shows a DG molecule without a phosphate group. (**D**) Typical binding curves of liposomes (PM) containing PC, PE, and PS, as well as glycolipids (DG) to histone H4 + H3 using Biacore X100. (**E**) A proposed model of interaction of histones with bilayers of lipid membrane to allow ions and proteins to flow across the cell membrane. (**F**) Potential membrane self-repairing mechanisms of the damage induced by pore-forming toxins.

The three-dimensional crystal structure of PLY has previously been resolved with modeling of its pore-structure on cell membranes [35]. However, the pore-structure model of extracellular histones on the cell membrane is unknown, although histone-mediated disruption of cell membrane is well documented [15,36]. Within the cell nucleus, the histone core interacts with double-stranded DNA via two layers of phosphate groups on phosphodiester backbones [37], which is similar to the bilayers of phosphate groups on the cell membrane (Figure 5A,B). SPR analysis demonstrates that phospholipids (PC, PE, and PS) with phosphate groups (PM) bind to histones, but glycolipids without phosphate group (DG) do not (Figure 5C,D). This suggests that extracellular histones bind the lipid bilayer of cell membranes via phosphate groups in the same way as the histone-core interaction with double-stranded DNA in nucleosomes [37]. However, how histones form pores on cell membranes is still not clear. Figure 5E proposes a model of histone integration into the bilayers of cell membranes. In this way, the Ca^2+^ influx will trigger the membrane self-repairing processes, including endosome formation to isolate the pores. Then, the endosome could be degraded inside the cell or released in the form of exosomes to the extracellular space (Figure 5F) to facilitate the restoration of cell function. This rapid self-repair mechanism to restore cell function compliments clinical observation of timely and complete recovery of multiple organ function after sepsis in the majority of cases.

## 4. Discussion

Membrane integrity is important to cellular function [38]. The disruption of membrane integrity by pore-forming toxins can be an important pathogenic mechanism in multiple organ injury, particularly in patients with sepsis, due to the possible coexistence of both endogenous and exogenous pore-forming toxins. In this study, we demonstrate that extracellular histones bind to phospholipids to form pores on cell membrane. In vitro, both extracellular histones and PLY bind membranes of cells derived from different organs and cause Ca^2+^ influx and leakage of specific biomarkers. This was also demonstrated in vivo using histone and PLY-infusion mouse models. The association of circulating histones with MODS has been demonstrated using both severe trauma and sepsis models with anti-histone intervention, as well as in a cohort of sepsis patients. The roles of exogenous pore-forming toxin, PLY, were explored by deletion of the PLY gene in D39 bacteria and demonstrated that PLY is not essential to sepsis development in mice, but increases organ injury and enhances lethality. These findings propose an important mechanism of MODS in sepsis and other critical illnesses.

It is known that extracellular histones are procoagulant and involved in the coagulopathy of sepsis via endothelial damage, platelet activation, thrombin generation, and affecting regulatory pathways, such as protein C, thrombomodulin, and fibrinolysis [11,14,39,40]. Coagulopathy is a major pathogenic factor that significantly contributes to microcirculatory impairment and MODS in many critical illnesses [41,42]. It is difficult to rectify whether coagulopathy or cytotoxicity contribute more to MODS. In general, non-survivors often develop shock and/or severe coagulopathy. It is fully justifiable that current treatments mainly focus on the correction of shock, coagulopathy, and poor tissue perfusion [43]. Clinical observation shows that, although a small fraction of patients who survive MODS may still experience neurological problems, recurrent infection, and deterioration of underlying diseases [44], full recovery of organ function is typically observed in the majority of patients. In this study, we showed that the SOFA scores of patients without shock and/or DIC were still correlated to the levels of circulating histones, strongly indicating that the pore-forming property of histones plays important roles in MODS in the subgroup of patients.

Pore-forming toxins can non-selectively bind plasma membranes of any cell type in contact [15,22]. Once these toxins enter circulation, endothelial cells will likely be the primary targets, leading to endothelial barrier disruption [15]. This might lead to the exposure of smooth muscle cells to toxins and cause Ca^2+^ overload, thereby potentially disrupting blood pressure and perfusion regulatory control, leading to septic shock [45]. Once parenchymal cells of different organs are exposed to pore-forming toxins, both non-specific (LDH) and cell-specific biomarkers (ALT, AST from liver cells, cTnI from cardiomyocytes) will be released into circulation. Unlike complement attack, the affected cells rarely lyse unless exposed to high concentrations of these toxins. The major pathological factor is more likely the resultant Ca^2+^ overload, which stresses cells and would affect cellular function [22,38], particularly in exciting cells, such as cardiomyocytes. This is consistent with the clinical observation that patients with severe sepsis frequently showed cardiac events, which could lead to premature death [46,47]. Many other factors also contribute to MODS, such as mitochondrial dysfunction [48] and injury to specific cells, including alveolar epithelial type II cells [49] and cardiomyocytes [50]. These pathological processes may also involve the disturbance of Ca^2+^ homeostasis.

Ca^2+^ influx is also a signal of plasma membrane damage of cells and will initiate the self-repair process [51,52], a central biological process for maintaining cellular homeostasis [29]. Although the detailed molecular mechanism of membrane self-repair is still not fully elucidated, vesicle trafficking, exocytosis, and endocytosis to remove the damaged membrane may all be involved [29]. The self-repair of cell membranes may be an important mechanism for the reversible processes in these survived patients to gain a full recovery of cellular and organ function.

The limitation of this study is the lack of available assays to monitor circulating PLY. However, using PLN-A strain without PLY and parental strain D39 with PLY to infect mice demonstrated that bacteria with PLY caused higher levels of ALT and LDH release into circulation and significantly reduced the survival time of mice. These data indirectly support that PLY-induced pore formation plays important roles in multiple organ injury and dysfunction in vivo. The co-existence of both endogenous and exogenous toxins may synergistically deteriorate MODS and accelerate the progression of lethal diseases. In our previous publications, our focuses were mainly on histone-induced coagulopathy and subsequent organ injury, as well as PLY-induced cardiomyocyte dysfunction.

This work focused on the importance of pore-forming properties of these mediators in order to better understand the pathophysiology of MODS. In reality, over 60% of patients with MODS are not complicated by shock or DIC. Therefore, a strategic focus towards reducing the cytotoxicity of pore-forming toxins, including accelerating membrane repairing, as well as reducing Ca^2+^ overload and its harmful effects, might achieve a rapid and improved recovery of organ functions in those patients. This strategy may also hold significant clinical value in patients with shock and DIC by reducing the severity of organ injury and MODS and increasing survival.

## Figures and Tables

**Table 1:** Patient Summary Characteristics.

	Total Patients (*n* = 98)
Age, median (IQR)	63.0 (49.8–72.3)
Gender, male, *n* (%)	57.0 (58.5)
Ethnicity, white, *n* (%)	78.0 (79.6)
Admission diagnosis, *n* (%)	
Sepsis	51.0 (52.0)
Respiratory sepsis	19.0 (19.4)
Abdominal sepsis	13.0 (13.3)
Urological sepsis	7.0 (7.1)
Cardiovascular sepsis	4.0 (4.1)
Other septic location	8.0 (8.2)
Trauma	13.0 (13.3)
Cardiovascular	13.0 (13.3)
Respiratory	11.0 (11.2)
Gastrointestinal	6.0 (6.1)
Central nervous system	3.0 (3.1)
Renal	1.0 (1.0)
APACHE II score, median (IQR)	22.0 (14.8–28.0)
SOFA score, median (IQR)	
Day 1	8.0 (4.0–11.0)
Day 2	8.0 (5.0–11.5)
Day 3	8.0 (5.0–11.0)
Day 4	8.0 (4.0–10.0)
Organ support, *n* (%)	
Respiratory	62.0 (63.3)
Cardiovascular	96.0 (98.0)
Renal	27.0 (27.6)
Organ support (Days), median (IQR)	
Respiratory	2.5 (0.0–11.0)
Cardiovascular	6.5 (4.0–14.0)
Renal	0.0 (0.0–2.0)
Length of stay (Days), median (IQR)	7.5 (5.0–17.0)
Mortality, *n* (%)	20.0 (20.4)

IQR; Interquartile range, APACHE II score; Acute Physiology and Chronic Health Evaluation II score, SOFA score; Sequential Organ Failure Assessment score.

**Table 2:** Spearmen’s rank correlation coefficient of circulating histones and organ injury scores in critically ill patients.

	Total (*n* = 98)		Non-Shock and Non-DIC (*n* = 64)	
	Histones		Histones	
SOFA score	Day 1	Day 2	Day 1	Day 2
Day 1	0.564	0.614	0.365	0.434
Day 2	0.559	0.598	0.428	0.386
Day 3	0.671	0.704	0.567	0.532
Day 4	0.643	0.621	0.506	0.415

This table shows the Spearmen’s rank correlation efficiency (*r* values) between daily circulating histones (Day 1, Day 2) and daily SOFA scores (Days 1–4) performed in the total cohort (*n* = 98) and in patients without shock and DIC (*n* = 64).

**Table 3:** Correlation of circulating histones to organ injury markers in mouse models.

Models	Organ Injury Markers	*n* *	*r*
Trauma	LDH	32	0.633
	ALT	27	0.679
	cTnI	21	0.591
	Lung injury scores	17	0.552
Sepsis	LDH	27	0.458
	ALT	22	0.533
	cTnI	18	0.476
	Lung injury scores	22	0.601

* *n* = number of mice with the data for r value calculation.

## Data Availability

The data presented in this study are available on reasonable request from the corresponding authors. Clinical data are restricted due to ethical considerations.
